# RNA interference analysis of potential functions of cyclin A in the reproductive development of male oriental river prawns (*Macrobrachium nipponense*)

**DOI:** 10.3389/fgene.2022.1053826

**Published:** 2022-11-17

**Authors:** Wenyi Zhang, Yiwei Xiong, Pengchao Wang, Tianyong Chen, Sufei Jiang, Hui Qiao, Yongsheng Gong, Yan Wu, Shubo Jin, Hongtuo Fu

**Affiliations:** ^1^ Key Laboratory of Freshwater Fisheries and Germplasm Resources Utilization, Ministry of Agriculture and Rural Affairs, Freshwater Fisheries Research Center, Chinese Academy of Fishery Sciences, Wuxi, China; ^2^ Wuxi Fisheries College, Nanjing Agricultural University, Wuxi, China; ^3^ National Demonstration Center for Experimental Fisheries Science Education, Shanghai Ocean University, Shanghai, China

**Keywords:** *Macrobrachium* nipponense, cyclin A, RNA interference, insulin-like androgenic gland hormone, male reproduction

## Abstract

Cyclin A (*CycA*) plays essential roles in regulating multiple steps of the cell cycle, and it affects gonad development in mammals and invertebrates. Previous RNA interference (RNAi) analysis revealed that knocking-down the expression of *CycA* in female oriental river prawns (*Macrobrachium nipponense*) inhibited ovarian development. *CycA* was also predicted to have regulatory roles in reproductive development of male *M. nipponense* based on significant changes of *Mn-CycA* expression after eyestalk ablation. The goal of this study was to investigate the potential functions of *CycA* in the reproductive development of male *M. nipponense* using RNAi and histological observations. Quantitative real-time PCR analysis revealed that both single-side and double-side eyestalk ablation stimulated the expressions of *Mn-CycA*, and the expression was higher in prawns with double-side eyestalk ablation (*p* < 0.05). *Mn-CycA* expression was significantly higher in the testis and androgenic gland during the reproductive season than during the non-reproductive season (*p* < 0.05). In the RNAi analysis, *Mn-CycA* expression significantly decreased after prawns were injected with *dsCycA*, and the expression of *insulin-like androgenic gland hormone* (*Mn-IAG*) also decreased as *Mn-CycA* expression decreased. This result indicated that *CycA* positively regulated the expression of *IAG* in *M. nipponense*. Histological observations revealed that the number of sperm decreased dramatically to <5% of the total cells in the testis of the *dsCycA*-treated group compared to that of control group on day 14, indicating that knockdown of *Mn-CycA* expression inhibited testis development by affecting the expression of *Mn-IAG* in *M. nipponense*. These results highlighted the functions of *CycA* in male reproductive development of *M. nipponense*, which can be applied to future studies of male reproduction in other crustacean species.

## Introduction

The oriental river prawn (*Macrobrachium nipponense*) (Crustacea; Decapoda; Palaemonidae) is widely distributed in China and other Asian countries, where it inhabits freshwater and low salinity estuarine regions (Jin S. B. et al., 2021). It is an economically important freshwater prawn in China, with annual aquaculture production of 225,321 metric tons in 2019 (Zhang et al., 2020). Previous histological observations revealed that newly hatched male and female *M. nipponense* can reach sexual maturity within 40 days after hatching during the reproductive season (Jin et al., 2016). The rapid gonad development of hatchlings leads to inbreeding between young prawns, resulting in mating and propagation of multiple generations in the same ponds. This leads to prawns with smaller market size, thereby restricting the sustainable development of the *M. nipponense* industry (Jin et al., 2022). Thus, the long-term goal of genetic improvements in *M. nipponense* is to regulate the process of gonad development.

Jin S. B. et al. (2021) previously reported that the expression of *Mn-Cyclin A* (*Mn-CycA*) increased after the ablation of eyestalks from male *M. nipponense*. They also found that eyestalk ablation promoted testis development by stimulating the expression of insulin-like androgenic gland hormone (IAG) (Jin S. B. et al., 2021; Jin et al., 2021b). The negative regulatory relationship between *CycA* and eyestalks suggested that *CycA* may be involved in regulating male reproductive development in *M. nipponense*.

Cyclins are synthesized and degraded in a cell cycle-dependent fashion. They are involved in cell cycle regulation through the formation of integral regulatory subunits of protein kinase complexes with cyclin-dependent kinases (*CDKs*). *CycA* is involved in the regulation of multiple steps in the cell cycle, including the S and G2/M phases of *Saccharomyces cerevisiae* and animal cells (Tarn and Lai, 2011). *CycA* binds with *CDK2* during G1/S conversion to promote replication of meiotic chromosomes and to enhance transcriptional activity of genes encoding estrogen and progesterone receptors (Rogatsky et al., 1986; Narayanan et al., 2005). *CycA* also forms a complex with *CDK1* during the late S phase, and the compound activates and stabilizes *cyclin B* and *CDK1* (Minshull et al., 1990; Lees and Harlow, 1993; Li et al., 2004).

The full-length cDNA sequence of *Mn-CycA* has been submitted to NCBI with the accession number of MT802360.1. In a previous study of *M. nipponense*, *CycA* expression was highest in the ovary and then testis, and the values were significantly higher than those of the other tested tissues, indicating the *CycA* may play regulatory roles in gonad development in *M. nipponense* (Zhou Z. Y. et al., 2021). RNA interference (RNAi) analysis in female *M. nipponense* revealed that knockdown of *CycA* expression inhibited ovarian development (Zhou Z. Y. et al., 2021). However, the regulatory roles of *CycA* on the reproductive development of male *M. nipponense* still needs to be investigated.

The goal of this study was to analyze the potential functions of *CycA* in male reproductive development of *M*. *nipponense* using quantitative real-time PCR (qPCR), *in situ* hybridization, RNAi, and histological observations. The results of this study highlighted the functions of *CycA* in *M*. *nipponense*, providing a basis for further studies of the mechanism of male reproduction in other crustacean species.

## Materials and methods

### Ethics statement

We obtained permission from the Institutional Animal Care and Use Ethics Committee of the Freshwater Fisheries Research Center, Chinese Academy of Fishery Sciences (Wuxi, China) to conduct all experiments involving *M. nipponense*.

The animal study was reviewed and approved by We obtained the permission from the Institutional Animal Care and Use Ethics Committee of the Freshwater Fisheries Research Center, Chinese Academy of Fishery Sciences (Wuxi, China) for all experiments involving M. nipponense. Written informed consent was obtained from the owners for the participation of their animals in this study.

### The qPCR analysis

The qPCR was used to measure the relative mRNA expression of *Mn-CycA* in the testis after the ablation of eyestalks from male *M. nipponense* and the expression in the testis and androgenic gland of male prawns collected during the reproductive and non-reproductive seasons. Three hundred healthy male *M*. *nipponense* (body weight, 3.47–3.89 g) were collected from the Dapu *M. nipponense* Breeding Base in Wuxi, China (120°13′44″E, 31°28′ 22″N). The prawns were randomly divided into three groups: control (CG), single-side eyestalk ablation (SS), and double-side eyestalk ablation (DS). They were transferred into three 500 L tanks and maintained in aerated freshwater for 3 days before eyestalk ablation. The testes were collected from prawns in each group on days 1, 4, and 7 and immediately preserved in liquid nitrogen until used for qPCR analysis. Testes from five different prawns were pooled to form a biological replicate, and three biological replicates were analyzed for each time point.

For the temporal comparison, testis and androgenic gland were collected from male *M. nipponense* during the reproductive season in July, when the water temperature was 30 ± 2 °C and the illumination time was ≥16 h. Tissues were also collected during the non-reproductive season in January, when the water temperature was 13 ± 2 °C and illumination time was ≤12 h. Five prawns were pooled to form one biological replicate, and three biological replicates were analyzed.

Details about the procedures used for RNA isolation and cDNA synthesis were described in previous studies (Jin et al., 2014; Jin et al., 2018). Briefly, total RNA from each tissue was extracted using the UNlQ-10 Column TRIzol Total RNA Isolation Kit (Sangon, Shanghai, China) following the manufacturer’s protocol. The PrimeScript™ RT Reagent Kit (Takara, Shiga, Japan) was used to synthesize the cDNA template according to the manufacturer’s instructions. The UltraSYBR Mixture (CWBIO, Beijing, China) was used to determine the expression level of *Mn-CycA* in each tissue. All of the SYBR Green RT-qPCR assays were performed using the Bio-Rad iCycler iQ5 Real-Time PCR System (Bio-Rad, Hercules, CA, United States ), and the qPCR reaction was run for three technical replicates for each tissue. Table 1 lists the primers used for qPCR analysis. Eukaryotic translation initiation factor 5A was used as the reference gene (Hu et al., 2018). The relative mRNA expressions of *Mn-CycA* were calculated based on the 2^−ΔΔCT^ comparative CT method.

**TABLE 1:** universal and specific primers used in this study.

Primer name	Nucleotide sequence (5′→3′)	Purpose
CycA-RTF	TGC​CCA​AAC​CAG​AAG​TCA​TTT​TC	FWD primer for Ferritin expression
CycA-RTR	TAT​GCT​TGG​ACC​GAT​GTC​TTC​AA	RVS primer for Ferritin expression
CycA anti-sense Probe	CCA​GAG​CAG​CGA​AGC​AGG​GTG​TCA​GCT​ATG​AAA​C	Probe for Ferritin ISH analysis
CycA sense Probe	GTT​TCA​TAG​CTG​ACA​CCC​TGC​TTC​GCT​GCT​CTG​G	Probe for Ferritin ISH analysis
CycA RNAi-F	TAA​TAC​GAC​TCA​CTA​TAG​GGA​AAT​GTT​TGC​CCA​AAC​CAG​A	FWD primer for RNAi analysis
CycA RNAi-R	TAA​TAC​GAC​TCA​CTA​TAG​GGA​TGC​TTG​GAC​CGA​TGT​CTT​C	RVS primer for RNAi analysis
EIF-F	CAT​GGA​TGT​ACC​TGT​GGT​GAA​AC	FWD primer for EIF expression
EIF-R	CTG​TCA​GCA​GAA​GGT​CCT​CAT​TA	RVS primer for EIF expression

### *In situ* hybridization

*In situ* hybridization was used to determine the mRNA locations of *Mn-CycA* in the testis and androgenic gland in shrimp collected during the reproductive season in July. The tissues were fixed in paraformaldehyde (4%) (Sangon, Shanghai, China), and then they were embedded in paraffin and sliced into 4 µm thick sections for the analysis. Details about the primer design and *in situ* hybridization can be found in previous reports (Jin et al., 2018; Li et al., 2018; Jin et al., 2022). Briefly, the specific anti-sense (experimental group) and sense (control group) probes were designed using Primer5 software based on the cDNA sequence of *Mn-CycA*. The specific primers with a digoxigenin (DIG) tag were synthesized by Shanghai Sangon Biotech Company (Table 1). The Zytofast PLUS CISH Implementation Kit (Zyto Vision GmBH, Bremerhaven, Germany) was used to perform the *in situ* hybridization analysis of the sectioned tissues following the manufacturer’s protocol. Slides were examined under a light microscope (Olympus Corporation, Tokyo, Japan).

### RNAi analysis

The potential function of *CycA* in reproductive development of male *M. nipponense* was investigated by RNAi. Snap Dragon tools (http://www.flyrnai.org/cgibin/RNAifind_primers.pl) was used to design the specific RNAi primer with a T7 promoter site based on the open reading frame of *Mn-CycA* (Table 1). The *Mn-CycA* dsRNA (*dsCycA*) was synthesized using the Transcript Aid™ T7 High Yield Transcription kit (Fermentas, Waltham, MA, United States ) following the manufacturer’s protocol. The green fluorescent protein dsRNA (*dsGFP*) was also synthesized and used as the negative control (Zhang et al., 2016).

Six hundred male *M*. *nipponense* were collected from the Dapu *M. nipponense* Breeding Base approximately 5 months after hatching (body weight, 3.15–4.37 g) and randomly divided into the *dsCycA* group (RNAi) and *dsGFP* group (control). The injected dose of *dsCycA* and *dsGFP* was 4 μg/g (Jiang et al., 2014; Li et al., 2018). Seven days after the first injection, 4 μg/g of *dsCycA* or *dsGFP* were injected again. Androgenic gland samples were collected from both the control group and the RNAi group on days 1, 7, and 14 after *dsGFP* or *dsCycA* injection. Androgenic gland samples from five different prawns at each time point were collected and pooled to form a biological replicate, and three biological replicates were tested. The *Mn-CycA* mRNA expression in the samples was measured by qPCR in order to confirm the silencing efficiency. The mRNA expression of *Mn-IAG* was also measured using the same cDNA templates to evaluate the regulatory relationship between *CycA* and *IAG* in *M. nipponense*.

### Haematoxylin and eosin (HE) staining

Morphological differences in the testis between prawns in the control and RNAi groups were assessed by HE staining following previously reported procedures (ShangGuan et al., 1991; Ma et al., 2006). Briefly, the tissues were dehydrated in different concentrations of ethanol and then embedded in xylene and wax. The embedded tissues were sectioned to a thickness of 5 µm using a microtome (Leica, Wetzlar, Germany). The sectioned tissues were placed on a slide and stained with HE for 3–8 min. The slides were observed under an Olympus SZX16 microscope (Olympus Corporation, Tokyo, Japan).

### Statistical analysis

All statistical analyses were conducted using SPSS Statistics 23.0 (IBM, Armonk, NY, United States ). The independent samples *t*-test was used to evaluate differences between the control and RNAi groups on the same day. Statistical differences were also identified by analysis of variance followed by least significant difference and Duncan’s multiple range tests. Quantitative data were expressed as mean ± standard deviation. A *p*-value < 0.05 was considered to be statistically significant.

## Results

### The qPCR analysis

**FIGURE 1:**
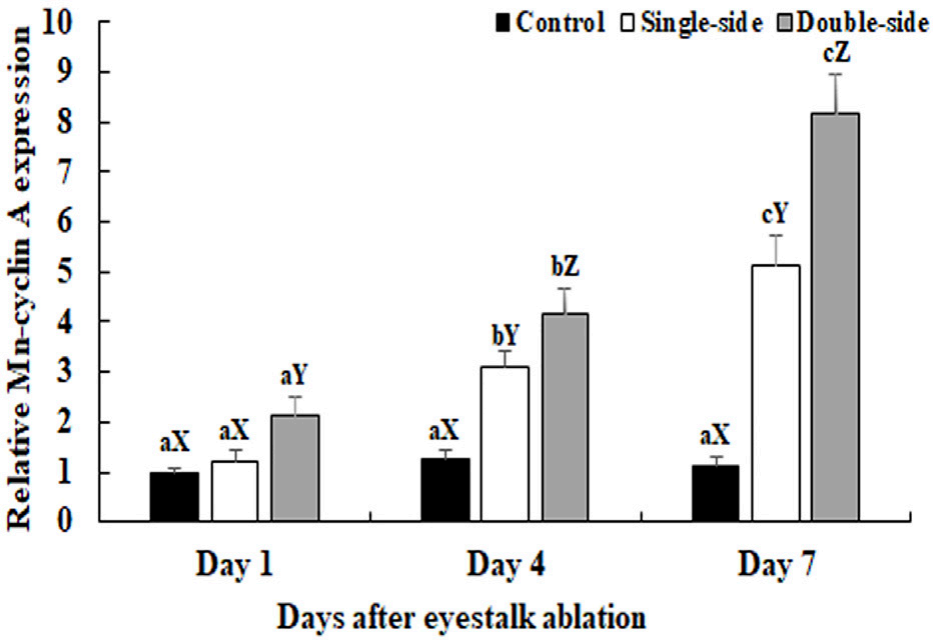
Measurement of the *Mn*-*CycA* expressions after the ablations of single-side and double-side eyestalk from male *M. nipponense* by qPCR. The amount of Mn-CycA mRNA was normalized to the EIF transcript level. Data are shown as mean ± SD (standard deviation) of tissues from three separate individuals. Lowercases indicate expression difference between different days in the same group (*p* < 0.05). Capital letters indicated the expression difference between different groups at the same day (*p* < 0.05).

**FIGURE 2:**
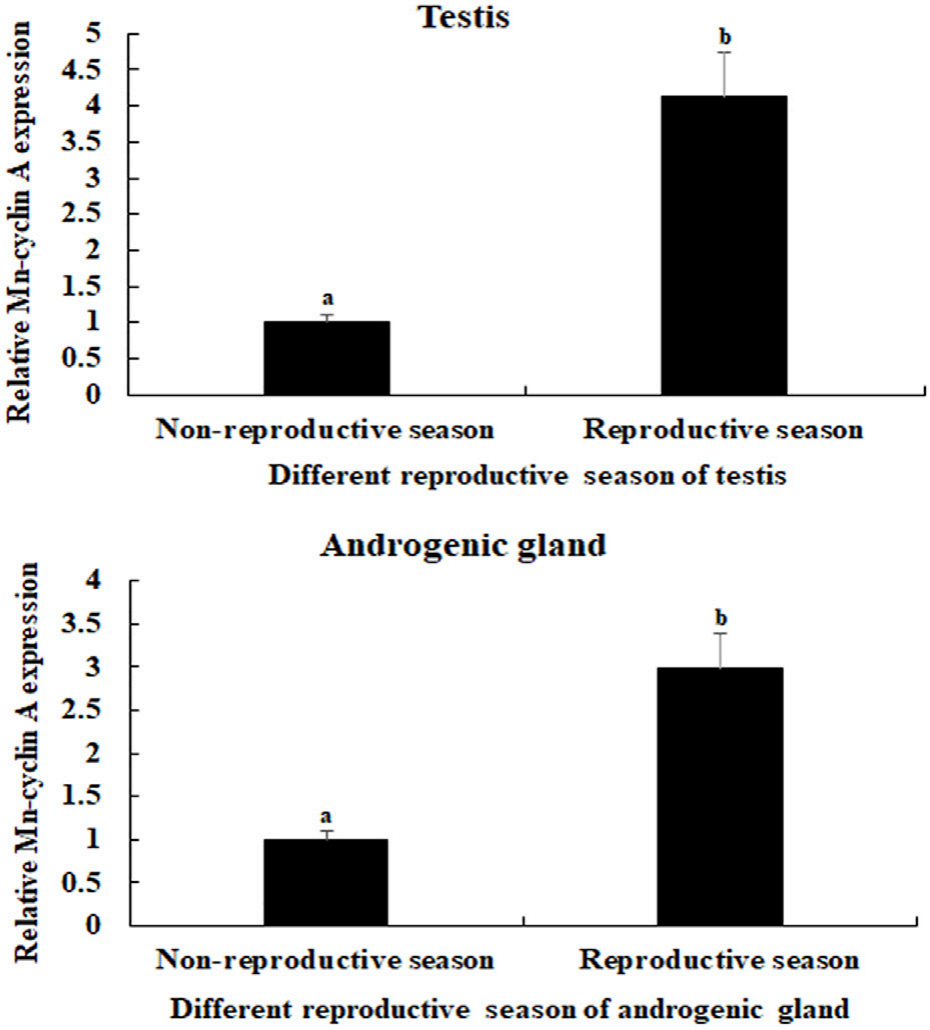
Measurement of the *Mn*-*CycA* expression in the testis and androgenic gland taken from different reproductive season. The amount of Mn-CycA mRNA was normalized to the EIF transcript level. Data are shown as mean ± SD (standard deviation) of tissues from three separate individuals. Lowercases indicate expression difference between different samples. **(A)** *Mn-CycA* expression in the testes taken from different reproductive season. **(B)** *Mn-CycA* expression in the androgenic gland taken from different reproductive season.

The physiological functions of a gene can be preliminary reflected by tissues distribution. The mRNA expression of *Mn-CycA* did not differ significantly in the testes between different days in control prawns (*p* > 0.05) (Figure 1). However, both single-side and double-side eyestalk ablation stimulated the mRNA expression of *Mn-CycA*. Expression continuously increased from day 1 to day 7, when the expression levels after single-side and double-side eyestalk ablation were 5.12-fold and 8.17-fold higher than that of day 1 in control prawns, respectively (*p* < 0.05). Furthermore, *Mn-CycA* expression in the prawns with double-side ablation was significantly higher than those of the control and single-side ablation groups on the same day after eyestalk ablation (*p* < 0.05). The qPCR analysis also showed that *Mn-CycA* expression in the testis and androgenic gland were 4.12-fold and 2.98-fold higher, respectively, during the reproductive season than during the non-reproductive season (*p* < 0.05) (Figure 2).

### *In situ* hybridization

**FIGURE 3:**
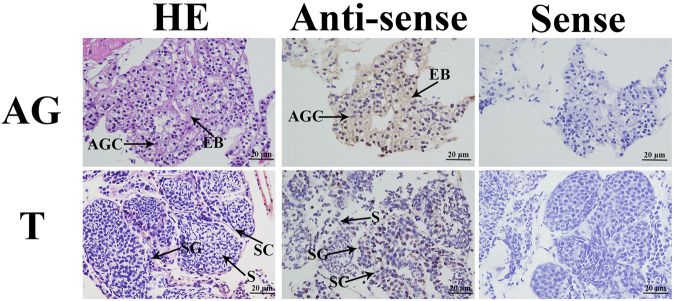
*In situ* hybridization analysis of *Mn*-*CycA* in the testis and androgenic gland taken from the reproductive season. SG: Spermatogonia; SC: spermatocyte; S: sperm; AGC: androgenic gland cells; EB: ejaculatory bulb. Scale bars = 20 μm.

In the *in situ* hybridization assay, HE staining revealed that the testis was composed of spermatogonia, spermatocytes, and sperm, and the androgenic gland includes the ejaculatory bulb and androgenic gland cells (Figure 3). No signal was directly observed in the androgenic gland cells, whereas DIG signals were observed in the ejaculatory bulb surrounding the androgenic gland cells. In addition, strong DIG signals were observed in the spermatogonia of the testis, whereas no signal was observed in the spermatocytes and sperm (Figure 3).

### RNAi analysis

**FIGURE 4:**
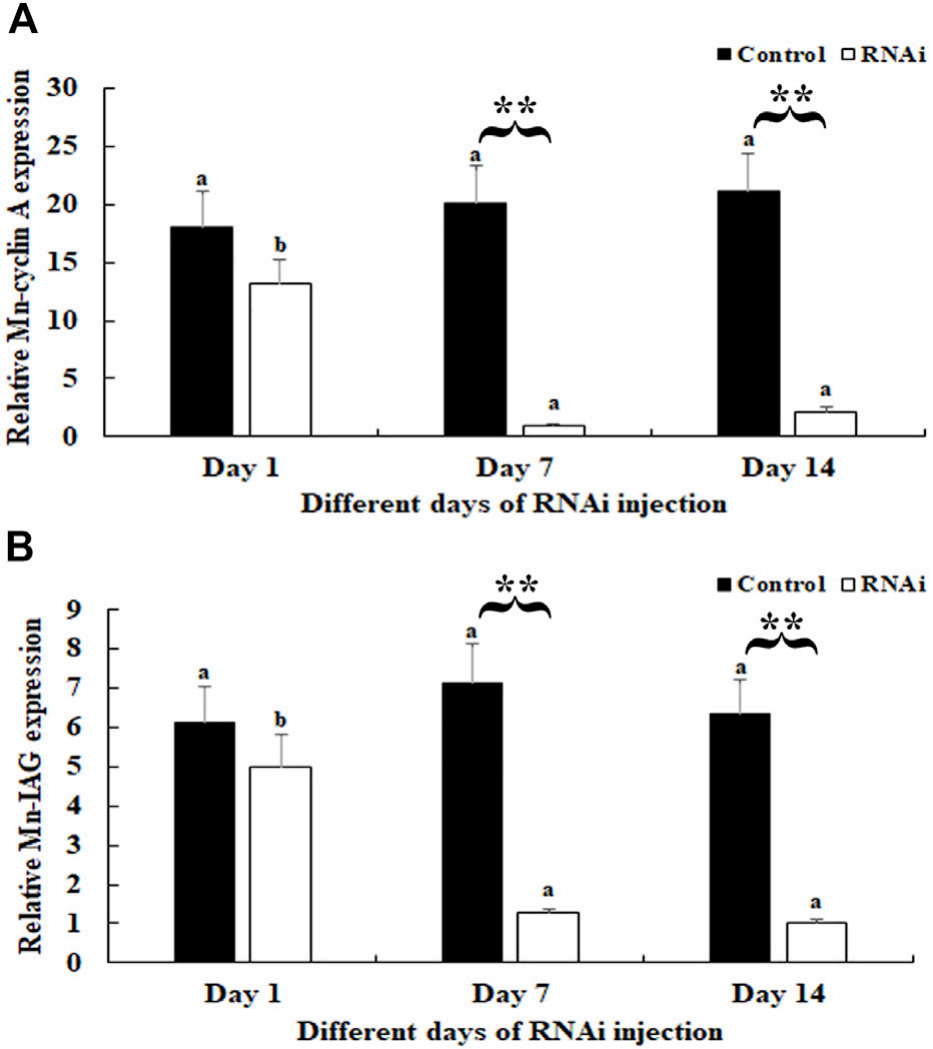
Measurement of *Mn*-*CycA* and *Mn-IAG* expression at different days after *dsCycA* and *dsGFP* injection. The amount of Mn-CycA and Mn-IAG mRNA was normalized to the EIF transcript level. Data are shown as mean ± SD (standard deviation) of tissues from three separate individuals. Lowercases indicated expression difference between different days after dsGFP and dsCycA injection. ** (*p* < 0.01) indicates significant expression difference between the dsCycA and dsGFP treated prawns at the sample day. **(A)** Measurement *of Mn-CycA* expression at different days after *dsGFP* and *dsCycA* injection. **(B)** Measurement of *Mn-IAG* expression at different days after *dsGFP* and *dsCycA* injection.

In the RNAi experiment, the mRNA expression of *Mn-CycA* in the androgenic gland did not differ significantly between different days after the *dsGFP* treatment, and this result was verified by qPCR analysis (*p* > 0.05). However, a decrease of >90% was detected on days 7 and 14 after *dsCycA* treatment compared to that of the *dsGFP*-injected group on the same day (*p* < 0.01) (Figure 4A). The expression of *Mn-IAG* did not differ significantly on day 1 after *dsCycA* injection compared to that of the *dsGFP* group (*p* > 0.05). However, it decreased by > 80% on days 7 and 14 in the *dsCycA*-injected group compared to the *dsGFP* group on the same day (*p* < 0.01) (Figure 4B).

### Histological observations

**FIGURE 5:**
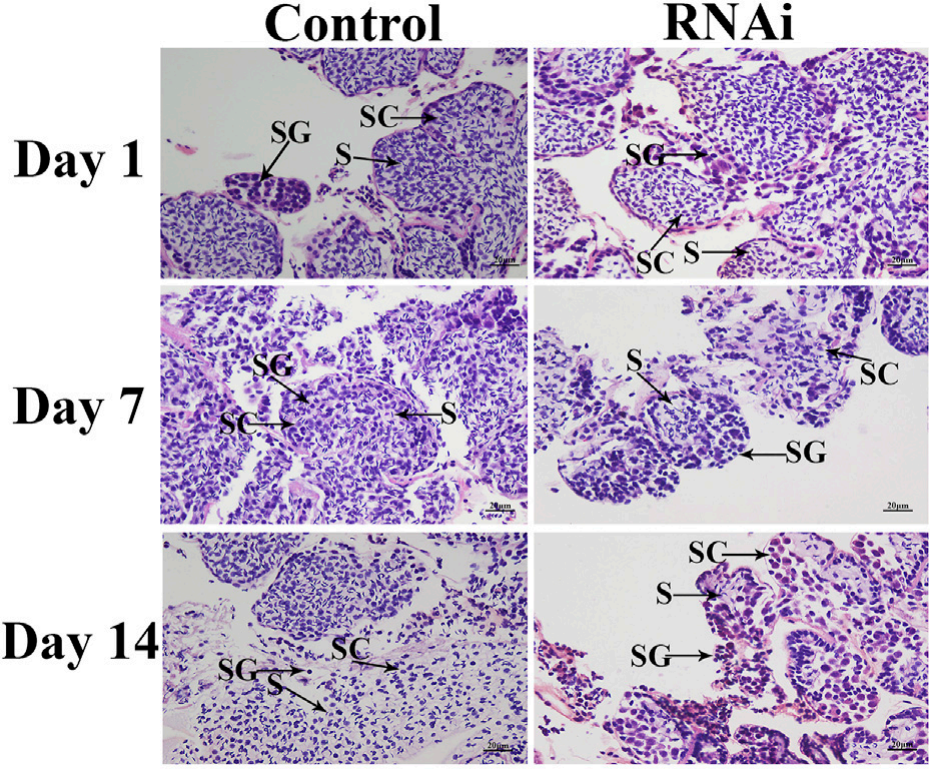
The histological observations of testis between dsCycA and dsGFP treated prawns. SG: Spermatogonia; SC: spermatocyte; S: sperm. Scale bars = 20 μm.

Histological observations revealed that the cell types did not differ on different days in the control group, and over 50% of the cells were sperm in the testis of *dsGFP*-treated prawns. In contrast, knockdown of the expression of *Mn-CycA* significantly decreased the number of sperm in the testis, and <5% cells were sperm on day 14 in the *dsCycA*-treated prawns (Figure 5).

## Discussion

Tarn and Lai (2011) reported that *CycA* regulates multiple steps of the cell cycle and plays a critical role in the S and G2/M phases in animal cells. A previous study of *CycA* in *M. nipponense* revealed that knockdown of *CycA* expression resulted in delayed ovarian development in *M. nipponense* (Zhou Z. Y. et al., 2021). In addition, Zhou Z. Y. et al. (2021) found that *CycA* expression was highest in the ovary and then the testis, and Jin S. B. et al. (2021) reported that its expression significantly increased in the androgenic gland at 7 days after eyestalk ablation from male *M. nipponense*. These results suggested that *CycA* was involved in the regulation of reproductive development in male *M. nipponense*, and in this study we further investigated the potential roles of *CycA* in this process.

To the best of our knowledge, we are the first to analyse the regulatory relationship between the eyestalk and *CycA* expression in a crustacean species. The qPCR analysis revealed that the expression of *Mn-CycA* significantly increased over time after both single-side and double-side eyestalk ablation, and expression was higher in the double-side eyestalk ablation group. This result indicated that double-side eyestalk ablation had a greater effect on promoting male reproductive development than single-side eyestalk ablation, which is consistent with results of previous studies (Jin S. B. et al., 2021; Jin et al., 2021b). The X-organ–SG complex is located in the eyestalk and is a principal neuroendocrine gland that stores and releases many neurosecretory hormones (Hopkins, 2012), including ion transport peptides, gonad-inhibiting hormone (*GIH*), crustacean hyperglycemic hormone (*CHH*), molt inhibiting hormone (*MIH*), and mandibular organ-inhibiting hormone. These hormones play essential roles in many biological functions of crustacean species (Santos et al., 1997; Almeida et al., 2004; Tiu and Chan, 2007; Sainz-Hernandez et al., 2008; Treerattrakool et al., 2011; Diarte-Plata et al., 2012; Pamuru et al., 2012; Salma et al., 2012; Revathi et al., 2013; Shen et al., 2013; Treerattrakool et al., 2013). In *M. nipponense*, *CHH* and *MIH* were reported to be involved in regulation of testis development and molting, respectively (Jin et al., 2013; Qiao et al., 2018). Additionally, the neurosecretory hormones secreted by eyestalks were found to have negative regulatory effects on both testis and ovarian development in *M. nipponense*. Qiao et al. (2015) showed that RNAi of *GIH* significantly promoted ovarian development in *M. nipponense*. Ablation of eyestalks from male *M. nipponense* stimulated the expression of *Mn-IAG* (Jin S. B. et al., 2021), with promoted testis development (Jin et al., 2021b). In the current study, the continuous increase of *Mn-CycA* expression after eyestalk ablation from male *M. nipponense* indicated that *CycA* may positively regulate reproductive development of male prawns. qPCR analysis also revealed higher *Mn-CycA* expression in the testis and androgenic gland during the reproductive season than during the non-reproductive season, which further supported the role of *CycA* in regulating reproductive development of male *M. nipponense*.

Prior to this study, *in situ* hybridization analysis of *CycA* had only been reported for female *M. nipponense*. Zhou Z. Y. et al. (2021) detected DIG signals of *Mn-CycA* throughout ovarian development, and they were mainly located in oogonia and oocytes. In male crustaceans, the androgenic gland and testis are the main reproductive organs (Sagi et al., 1986; Sagi and Cohen, 1990; Qiu et al., 1995). In the current study, DIG signals were observed in the ejaculatory bulb that surrounds the androgenic gland but not in androgenic gland cells, indicating that *CycA* did not directly involved in process of sex determination and differentiation in *M. nipponense*, while it played essential roles in the formation and maintenance of the normal structure of the androgenic gland (Jin et al., 2018; JinC et al., 2021; Jin et al., 2022). In the testis, DIG signals were only observed in the spermatogonia, indicating that *CycA* was involved in the process of spermatogenesis in *M. nipponense*, especially that of the spermatogonia proliferation (JinC et al., 2021; Jin et al., 2022).

The expression of *CycA* is known to affect the process of testis and ovarian development in many mammals and invertebrates. Liu et al. (1998) reported that targeted deletion of *CycA1* from male mice blocked spermatozoa development before the first meiosis, resulting in male infertility. Female fruit flies (*Drosophila*) can lay eggs after knockdown of the expressions of *CycA*, but the eggs cannot hatch. In addition, low expression of *CycA* can result in delayed rupture of the meiotic nuclear membrane and mislocalization of the middle of the spindle at the end of mitosis (Bergman et al., 2015; Bourouh et al., 2016). Sandra et al. (2012) found that the development of germinal vesicle rupture and G2/M was inhibited during meiotic phase I after injection of *CycA2* antibody into mouse oocytes during meiosis. Knockdown of *CycA2* expression in oocytes at meiotic stage II inhibited the separation of sister chromatids. In the silk moth *Bombyx mori*, ovarian development was blocked in the G1 phase after knockdown of the expression of *CycA* in ovarian cells, which effectively inhibited cell proliferation (Liu et al., 2015). Knockdown of the expression of *CycA* in female *M. nipponense* by RNAi resulted in delayed ovarian development (Zhou Z. Y. et al., 2021). In the present study, RNAi was also employed to investigate the potential functions of *CycA* in the reproduction of male *M. nipponense*. The injection of *dsCycA* resulted in a significant decrease of *Mn-CycA* and *Mn-IAG* expressions on days 7 and 14, indicating that the synthesized *dsCycA* efficiently knocked-down the expression of these genes and that *CycA* has a positive regulatory role in the expression of *IAG* in *M. nipponense*. *IAG* is the main gene expressed in the androgenic gland, and its regulatory effects on male differentiation and reproduction have been well studied in *Macrobrachium rosenbergii*, especially those related to spermatogenesis (Sagi et al., 1986; Sagi and Cohen, 1990; Ventura et al., 2012). Similar functions of *IAG* have been reported in many other crustacean species (Li et al., 2012; Huang et al., 2014; Ma et al., 2016; Zhou T. T. et al., 2021; Liu, 2021). Histological observations revealed that sperm were the main cell type in the testis of *dsGFP*-injected prawns but that they accounted for <5% of cells on day 14 after *dsCycA* injection. This result indicated that RNAi of *Mn-CycA* had a significant inhibitory role on testis development of *M. nipponense*, which confirmed the positive regulatory roles of *CycA* in the regulation of testis development in this prawn.

In conclusion, eyestalk ablation from male *M. nipponense* continuously stimulated the expression of *Mn-CycA*, and *Mn-CycA* expression was higher in the testis and androgenic gland during the reproductive season than during the non-reproductive season. Knockdown of the expression of *Mn-CycA* by RNAi in male *M. nipponense* revealed that *CycA* had a positive regulatory effect on testis development of this prawn, which was verified by histological observations of testis samples from *dsGFP-injected* and *dsCycA*-injected prawns. These results showed that *CycA* had an important role in the reproductive development of male *M. nipponense*, which can be applied to the development of techniques to regulate testis development in *M. nipponense*.

## Data Availability

The raw data supporting the conclusion of this article will be made available by the authors, without undue reservation.

## References

[B1] Almeida E. A. Petersen R. L. Andreatta E. R. Bainy A. C. (2004). Effects of captivity and eyestalk ablation on antioxidant status of shrimps (*Farfantepenaeus paulensis*). Aquaculture 238, 523–528. 10.1016/j.aquaculture.2004.04.010

[B2] Bergman Z. J. McLaurin J. D. Eritano A. S. Johnson B. M. Sims A. Q. Riggs B. (2015). Spatial reorganization of the endoplasmic reticulum during mitosis relies on mitotic kinase cyclin A in the early Drosophila embryo. PLoS ONE 10, e0117859. 10.1371/journal.pone.0117859 25689737 PMC4331435

[B3] Bourouh M. Dhaliwal R. Rana K. Sinha S. Guo Z. Swan A. (2016). Distinct and overlapping requirements for cyclins A, B, and B3 in drosophila female meiosis. G3 6, 3711–3724. 10.1534/g3.116.033050 27652889 PMC5100870

[B4] Diarte-Plata G. Sainz-Hernández J. C. Aguiñaga-Cruz J. A. Fierro-Coronado J. A. Polanco-Torres A. Puente-Palazuelos C. (2012). Eyestalk ablation procedures tominimize pain in the freshwater prawn Macrobrachium Americanum. Appl. Anim. Behav. Sci. 140, 172–178. 10.1016/j.applanim.2012.06.002

[B5] Hopkins P. M. (2012). The eyes have it: A brief history of crustacean neuroendocrinology. Gen. Comp. Endocrinol. 175, 357–366. 10.1016/j.ygcen.2011.12.002 22197211

[B6] Hu Y. N. Fu H. T. Qiao H. Sun S. M. Zhang W. Y. Jin S. B. (2018). Validation and evaluation of reference genes for Quantitative real-time PCR in *Macrobrachium nipponense* . Int. J. Mol. Sci. 19, 2258. 10.3390/ijms19082258 30071669 PMC6121487

[B7] Huang X. S. Ye H. H. Huang H. Y. Yang Y. N. Gong J. (2014). An insulin-like androgenic gland hormone gene in the mud crab, Scylla paramamosain, extensively expressed and involved in the processes of growth and female reproduction. Gen. Comp. Endocrinol. 204, 229–238. 10.1016/j.ygcen.2014.06.002 24929228

[B8] Jiang F. W. Fu H. T. Qiao H. Zhang W. Y. Jiang S. F. Xiong Y. W. (2014). The RNA interference regularity of transformer-2 gene of oriental river prawn *Macrobrachium nipponense* . Chin. Agricul. Sci. Bul. 30 (32), 32–37.

[B9] Jin S. B. Fu H. T. Jiang S. F. Xiong Y. W. Sun S. M. Qiao H. (2018). Molecular cloning, expression, and *in situ* hybridization analysis of forkhead box protein L2 during development in Macrobrachium nipponense. J. World Aquac. Soc. 49 (10), 429–440. 10.1111/jwas.12510

[B10] Jin S. B. Fu Y. Hu Y. N. Fu H. T. Jiang S. F. Xiong Y. W. (2021a). Identification of candidate genes from androgenic gland in *Macrobrachium nipponense* regulated by eyestalk ablation. Sci. Rep. 11, 19855. 10.1038/s41598-021-99022-4 34615913 PMC8494903

[B11] Jin S. B. Jiang S. F. Xiong Y. W. Qiao H. Sun S. M. Zhang W. Y. (2014). Molecular cloning of two tropomyosin family genes and expression analysis during development in oriental river prawn, Macrobrachium nipponense. Gene 546 (2), 390–397. 10.1016/j.gene.2014.05.014 24809964

[B12] Jin S. B. Wang N. Qiao H. Fu H. T. Wu Y. Gong Y. S. (2013). Molecular cloning and expression of a full-length cDNA encoding crustacean hyperglycemic hormone (CHH) in oriental river pawn (*Macrobrachium nipponense*). J. Fish. Sci. China 20, 82–92. 10.3724/sp.j.1118.2013.00082

[B13] Jin S. B. Zhang Y. Guan H. H. Fu H. T. Jiang S. F. Xiong Y. W. (2016). Histological observation of gonadal development during post-larva in oriental river prawn, *Macrobrachium nipponense* . Chin. J. Fish. 29, 11–16.

[B14] Jin S. Fu H. Jiang S. Xiong Y. Qiao H. Zhang W. (2022). RNA interference analysis reveals the positive regulatory role of ferritin in testis development in the oriental river prawn, *Macrobrachium nipponense* . Front. Physiol. 13, 805861. 10.3389/fphys.2022.805861 35250613 PMC8896479

[B15] Jin S. Fu Y. Hu Y. Fu H. Jiang S. Xiong Y. (2021b). Transcriptome profiling analysis of the testis after eyestalk ablation for selection of the candidate genes involved in the male sexual development in *Macrobrachium nipponense* . Front. Genet. 12, 675928. 10.3389/fgene.2021.675928 34135943 PMC8202825

[B16] Jin S. Hu Y. Fu H. Jiang S. Xiong Y. Qiao H. (2021). Identification and characterization of the pyruvate dehydrogenase E1 gene in the oriental river prawn, *Macrobrachium nipponense* . Front. Endocrinol. 12, 752501. 10.3389/fendo.2021.752501 PMC8591192 34790171

[B17] Lees E. M. Harlow E. (1993). Sequences within the conserved cyclin box of human cyclin A are sufficient for binding to and activation of cdc2 kinase. Mol. Cell. Biol. 13, 1194–1201. 10.1128/mcb.13.2.1194 8423786 PMC359004

[B18] Li C. J. Vassilev A. DePamphilis L. D. (2004). Role for Cdk1 (Cdc2)/Cyclin A in preventing the mammalian origin recognition complex’s largest subunit (Orc1) from binding to chromatin during mitosis. Mol. Cell. Biol. 24, 5875–5886. 10.1128/MCB.24.13.5875-5886.2004 15199143 PMC480893

[B19] Li F. Qiao H. Fu H. T. Sun S. M. Zhang W. Y. Jin S. B. (2018). Identification and characterization of opsin gene and its role in ovarian maturation in the oriental river prawn *Macrobrachium nipponense* . Comp. Biochem. Physiol. B Biochem. Mol. Biol. 218, 1–12. 10.1016/j.cbpb.2017.12.016 29309912

[B20] Li S. H. Li F. H. Sun Z. Xiang J. H. (2012). Two spliced variants of insulin-like androgenic gland hormone gene in the Chinese shrimp, Fenneropenaeus chinensis. Gen. Comp. Endocrinol. 177 (2), 246–255. 10.1016/j.ygcen.2012.04.010 22561290

[B21] Liu D. Matzuk M. M. Sung W. K. Guo Q. Wang P. Wolgemuth D. J. (1998). Cyclin A1 is required for meiosis in the male mouse. Nat. Genet. 20, 377–380. 10.1038/3855 9843212

[B22] Liu F. Shi W. Ye H. Liu A. Zhu Z. (2021). RNAi reveals role of insulin-like androgenic gland hormone 2 (IAG2) in sexual differentiation and growth in hermaphrodite shrimp. Front. Mar. Sci. 8, 666763. 10.3389/fmars.2021.666763

[B23] Liu L. Shen W. Bing L. I. Qin J. Youlan Y. U. Pengyu M. A. (2015). RNA interference impact on silkworm cyclin A gene and cell proliferation. J. Nanjing Agric. Univ. 38 (1), 168–171.

[B24] Ma K. Y. Li J. L. Qiu G. F. (2016). Identification of putative regulatory region of insulin-like androgenic gland hormone gene (IAG) in the prawn Macrobrachium nipponense and proteins that interact with IAG by using yeast two-hybrid system. Gen. Comp. Endocrinol. 229, 112–118. 10.1016/j.ygcen.2016.03.019 26979275

[B25] Ma X. K. Liu X. Z. Wen H. S. Xu Y. J. Zhang L. J. (2006). Histological observation on gonadal sex differentiation in *Cynoglossus semilaevis* Günther. Mar. Fish. Res. 27 (2), 55–61.

[B26] Minshull J. Golsteyn R. Hill C. S. Hunt T. (1990). The A- and B-type cyclin associated cdc2 kinases in Xenopus turn on and off at different times in the cell cycle. EMBO J. 9, 2865–2875. 10.1002/j.1460-2075.1990.tb07476.x 2143983 PMC551999

[B27] Narayanan R. Adigun A. A. Edwards D. P. Weigel N. L. (2005). Cyclin-dependent kinase activity is required for progesterone receptor function: Novel role for cyclin A/Cdk2 as a progesterone receptor coactivator. Mol. Cell. Biol. 25, 264–277. 10.1128/MCB.25.1.264-277.2005 15601848 PMC538783

[B28] Pamuru R. R. Rosen O. Manor R. Chung J. S. Zmora N. Glazer L. (2012). Stimulation of molt by RNA interference of the molt inhibiting hormone in the crayfish *Cherax quadricarinatus* . Gen. Comp. Endocrinol. 178, 227–236. 10.1016/j.ygcen.2012.05.007 22664421

[B29] Qiao H. Jiang F. W. Xiong Y. W. Jiang S. F. Fu H. T. Li F. (2018). Characterization, expression patterns of molt-inhibiting hormone gene of Macrobrachium nipponense and its roles in molting and growth. PloS one 13, e0198861. 10.1371/journal.pone.0198861 29889902 PMC5995357

[B30] Qiao H. Xiong Y. W. Zhang W. Y. Fu H. T. Jiang S. F. Sun S. M. (2015). Characterization, expression, and function analysis of gonad-inhibiting hormone in Oriental River prawn, *Macrobrachium nipponense* and its induced expression by temperature. Comp. Biochem. Physiol. A Mol. Integr. Physiol. 185, 1–8. 10.1016/j.cbpa.2015.03.005 25770669

[B31] Qiu G. F. Du N. S. Lai W. (1995). Studies on the male reproductive system of the freshwater prawn. Macrobrachium Nippon. J. Shanghai Fish. Univ. 4, 107–111.

[B32] Revathi P. Iyapparaj P. Vasanthi L. A. Jeyanthi S. Krishnan M. (2013). Impact of eyestalk ablation on the androgenic gland activity in the freshwater prawn *Macrobrachium rosenbergii* (De Man). World 5, 373–381.

[B33] Rogatsky I. Trowbridge J. M. Garabedian M. J. (1986). Potentiation of human estrogen receptor α transcriptional activation through phosphorylation of serines 104 and 106 by the Cyclin A-CDK2 complex. J. Biol. Chem. 274, 22296–22302. 10.1074/jbc.274.32.22296 10428798

[B34] Sagi A. Cohen D. (1990). Growth, maturation and progeny of sex-reversed *Macrobrachium rosenbetgii* males. World Aquacult 21 (4), 87–90.

[B35] Sagi A. Ra’anan Z. Cohen D. Wax Y. (1986). Production of *Macrobrachium rosenbetgii* in momosex population: Yield characteristes under intensive monoculture conditions in cages. Aquaculture 51, 265–275. 10.1016/0044-8486(86)90318-2

[B36] Sainz-Hernandez J. C. Racotta I. S. Dumas S. Hernandez-Lopez J. (2008). Effect of unilateral and bilateral eyestalk ablation in *Litopenaeus vannamei* male and female on several metabolic and immunologic variables. Aquaculture 283, 188–193. 10.1016/j.aquaculture.2008.07.002

[B37] Salma U. Uddowla M. H. Kim M. Kim J. M. Bo K. K. Baek H. J. (2012). Five hepatopancreatic and one epidermal chitinases from a pandalid shrimp (*Pandalopsis japonica*): Cloning and effects of eyestalk ablation on gene expression. Comp. Biochem. Physiol. B Biochem. Mol. Biol. 161, 197–207. 10.1016/j.cbpb.2011.11.005 22138334

[B38] Sandra A. T. Damien C. Lisa M. L. Ioanna L. Jean-Philippe C. Ahmed R. (2012). Cyclin A2 is required for sister chromatid segregation, but not separase control, in mouse oocyte meiosis. Cell Rep. 2, 1077–1087. 10.1016/j.celrep.2012.10.002 23122964

[B39] Santos E. A. Eduardo L. Nery M. Goncalves A. A. Keller R. (1997). Evidence for the involvement of the crustacean hyperglycemic hormone in the regulation of lipid metabolism. Physiol. Zool. 70, 415–420. 10.1086/515846 9237301

[B40] ShangGuan B. M. Liu Z. Z. Li S. Q. (1991). Histological studies on ovarian development in *Scylla serrata* . J. Fish. Sci. China. 15 (2), 96–103.

[B41] Shen H. Zhou X. Bai A. Ren X. Zhang Y. (2013). Ecdysone receptor gene from the freshwater prawn Macrobrachium nipponense: Identification of different splice variants and sexually dimorphic expression, fluctuation of expression in the molt cycle and effect of eyestalk ablation. Gen. Comp. Endocrinol. 193, 86–94. 10.1016/j.ygcen.2013.07.014 23899714

[B42] Tarn W. Y. Lai M. C. (2011). Translational control of cyclins. Cell Div. 6, 5. 10.1186/1747-1028-6-5 21314915 PMC3048474

[B43] Tiu S. H. K. Chan S. M. (2007). The use of recombinant protein and RNA interference approaches to study the reproductive functions of a gonad-stimulating hormone from the shrimp *Metapenaeus ensis* . FEBS J. 274, 4385–4395. 10.1111/j.1742-4658.2007.05968.x 17725713

[B44] Treerattrakool S. Chartthai C. Phromma-in N. Panyim S. Udomkit A. (2013). Silencing of gonad-inhibiting hormone gene expression in *Penaeus monodon* by feeding with GIH dsRNA-enriched Artemia. Aquaculture 404, 116–121. 10.1016/j.aquaculture.2013.04.024

[B45] Treerattrakool S. Panyim S. Udomkit A. (2011). Induction of ovarian maturation and spawning in *Penaeus monodon* broodstock by double-stranded RNA. Mar. Biotechnol. 13, 163–169. 10.1007/s10126-010-9276-0 20333425

[B46] Ventura T. Manor R. Aflalo E. D. Weil S. Rosen O. Sagi A. (2012). Timing sexual differentiation: Full functional sex reversal achieved through silencing of a single insulin-like gene in the prawn, *Macrobrachium rosenbergii* . Biol. Reprod. 86 (3), 90. 10.1095/biolreprod.111.097261 22133694

[B47] Zhang S. B. Jiang P. Wang Z. Q. Long S. R. Liu R. D. Zhang X. (2016). Dsrna-mediated silencing of nudix hydrolase in Trichinella spiralis inhibits the larval invasion and survival in mice. Exp. Parasitol. 162, 35–42. 10.1016/j.exppara.2016.01.005 26778819

[B48] Zhang X. L. Cui L. F. Li S. M. Liu X. Z. Han X. Jiang K. Y. (2020). Bureau of Fisheries, ministry of agriculture, P.R.C. Fisheries economic statistics. China Fishery Yearbook. Beijing China Agricultural Press, 24.

[B49] Zhou T. T. Wang W. Wang C. G. Sun C. B. Shi L. L. Chan S. F. (2021a). Insulin-like androgenic gland hormone from the shrimp fenneropenaeus merguiensis: Expression, gene organization and transcript variants. Gene 782, 145529. 10.1016/j.gene.2021.145529 33631246

[B50] Zhou Z. Y. Fu H. T. Jin S. B. Qiao H. Zhang W. Y. Jiang S. F. (2021b). Function analysis and molecular characterization of cyclin A in ovary development of oriental river prawn, *Macrobrachium nipponense* . Gene 788, 145583. 10.1016/j.gene.2021.145583 33753150

